# Evaluating physicians’ knowledge of assisted suicide and palliative sedation therapy in Austria: An exploratory survey study

**DOI:** 10.1007/s00508-025-02509-7

**Published:** 2025-03-04

**Authors:** Lukas Fischer, Nguyen-Son Le, Stefanie Kirchner, Angelika Feichtner, Angelina Falkner, Eva Katharina Masel, Jan Gärtner, Dietmar Weixler, Gudrun Kreye

**Affiliations:** 1 Karl Landsteiner University of Health Sciences, Krems, Austria; 2 Division of Palliative Care, Department of Internal Medicine 2, University Hospital Krems, Mitterweg 10, 3500 Krems an der Donau, Austria; 3 Public Mental Health Research Unit, Department of Social and Preventive Medicine, Center for Public Health, Medical University of Vienna, Vienna, Austria; 4 Austrian Association for Palliative Care, Vienna, Austria; 5 Department of Internal Medicine I, Clinical Division of Palliative Medicine, Medical University Vienna, Vienna, Austria; 6 Palliative Care Center Basel, Basel, Switzerland; 7 Department of Clinical Research, University Hospital Basel and University of Basel, Basel, Switzerland; 8 Department of Anesthesiology, Hospital Horn, Horn, Austria

**Keywords:** End-of-life decision-making, Medical ethics, Physician education, Legal framework in healthcare, Symptom management

## Abstract

**Background:**

Since January 2022, assisted suicide (AS) has been legal in Austria under specific conditions, allowing medical AS for individuals with incurable, life-ending diseases or severe, enduring illnesses that significantly impact their lives. Palliative sedation therapy (PST) involves the controlled use of drugs to induce unconsciousness and alleviate otherwise untreatable suffering. This study evaluates Austrian physicians’ knowledge of AS and PST, comparing the knowledge levels of palliative care (PC) and non-PC physicians.

**Methods:**

In this explorative study, an online survey was distributed primarily to physicians in Lower Austria through the State Healthcare Agency Lower Austria (Landesgesundheitsagentur Niederösterreich, LGA) and to PC physicians across Austria via the Austrian Association for Palliative Care (Österreichische Palliativgesellschaft, OPG). Questions in this survey focused on basic knowledge of the principles of AS and PST. Participants included physicians who have expertise in PC and physicians from other medical specialties.

**Results:**

In this study, 223 physicians completed the survey. The PC physicians displayed significantly more specialized training, with 74.2% holding a PC diploma compared to 17.9% of non-PC physicians (*p* < 0.001). Regarding PST, PC physicians were more likely to follow guidelines, using midazolam (97.8% vs. 77.6%, *p* < 0.001), propofol (56.2% vs. 38.1%, *p* = 0.009), and levomepromazine (23.6% vs. 11.2%, *p* = 0.016). In AS, PC physicians emphasized the decision-making capacity more frequently (*p* = 0.006) and were better informed about the legal requirements.

**Conclusion:**

Overall, Austrian physicians demonstrate insufficient knowledge of AS and PST, underlining the need for enhanced education on PC principles to ensure informed practice post-AS legalization. Nonetheless, PC physicians in Austria demonstrate superior adherence to guidelines in managing life-limiting conditions, PST and AS compared to non-PC physicians, highlighting the importance of specialized PC education.

## Introduction

Palliative care (PC) is an approach designed to improve the quality of life for patients and their families facing life-threatening illnesses. It focuses on the prevention and relief of suffering through early implementation and treatment of symptoms, such as pain and dyspnea, as well as addressing psychological, spiritual and social issues [1]. Timely integration of PC is essential when certain trigger points in the disease course are met [2].

In Austria, PC facilities serve incurably ill and dying individuals, aiming to improve their quality of life and facilitate discharge into familiar surroundings [3]. Care is provided by an interdisciplinary team addressing physical, emotional, social and spiritual needs, with involvement from volunteers; however, as societies evolve, the ethical, legal and clinical boundaries of care for terminally ill patients are increasingly being tested. This is particularly evident in Austria, where the Constitutional Court ruled in December 2020 that banning all forms of assisted suicide without exception was unconstitutional. This decision, which took effect in 2022, marked a significant shift in the legal landscape of self-determined death and raised complex questions about the interplay between assisted suicide and PC [4].

Despite the legalization of assisted suicide (AS) in Austria in 2022, empirical data on end of life decisions made by Austrian physicians are scarce. A 2020 study revealed that attending physicians felt the legal situation was unclear. It is recommended that physicians receive training regarding end of life choices and medical decisions [5].

The term AS must be clearly distinguished from palliative sedation therapy (PST), which does not intend to end life but to alleviate suffering [6, 7]. The PST includes a broad range of tasks aimed at relieving distress in terminally ill patients, focusing on resolving refractory symptoms at the end of life [8, 9]. Although PST provides benefits for patients, it is used hesitantly by physicians due to a lack of experience in defining the severity of symptoms requiring PST and insufficient knowledge about this form of therapy among patients, family members and healthcare providers [10].

The main goal of this study was to evaluate the current knowledge of PC physicians and non-PC physicians in Lower Austria regarding PC, PST and AS. We also sought to determine whether there is a difference in knowledge between PC physicians and physicians from other specialties.

## Material and methods

### Study design—Questionnaire

The study was designed as an explorative survey study. We utilized a structured questionnaire to gather data from medical professionals on their knowledge, perceptions, attitudes and practices related to PST and AS. Following an extensive review of the relevant literature, we developed a survey containing questions about PST and AS. Preliminary versions of the questionnaire were distributed to volunteers for testing.

The questionnaire was developed by a researcher with a PhD in public health, proficient in statistics and survey design, with a track record of expertise demonstrated through multiple publications. Additionally, three PC physicians with extensive clinical experience and expertise in all topics covered by the survey contributed to its development.

The questionnaire was piloted among colleagues to assess clarity and relevance. The pilot group included 8 PC physicians and 16 non-PC physicians. Feedback from this pilot testing was used to refine the questionnaire where necessary, ensuring its suitability for the target audience.

The final questionnaire was structured to gather comprehensive information through several sections. The questionnaire consisted of four sections: demographic data (age, gender, field of expertise, PC training and work experience), knowledge-based questions on PST and AS, perception and attitude questions about views on PST and AS and experience and practice questions regarding the application of PST practices, such as the frequency of palliative sedation, drug usage, supervision access and patient services. Correct answers and detailed content of the knowledge-based questions are available in the supplementary material.

Regarding knowledge-based questions, topics included PST and AS. The PST questions covered its definition (correct: relief from suffering with sedatives), guideline-recommended drugs (correct: midazolam, levomepromazine, propofol), indications (correct: severe symptoms) and authorized practitioners (correct: physicians). The AS questions examined qualifying conditions (correct: incurable disease, adult, free decision), Assisted Dying Act (Sterbeverfügungsgesetz, StVfG) requirements (correct: consult doctors/notary, 12-week wait), the legal waiting period (correct: 12 weeks, 2 weeks terminal), the performer (correct: patients), counselling information (correct: alternatives, medication, method, complications, refusal information), and preparation actions (correct: prepare infusion, dissolve powder, insert needle, document thoughts).

The questionnaire was administered online, with the link distributed primarily by the State Health Agency of Lower Austria (Landesgesundheitsagentur Niederösterreich, LGA), targeting hospitals and healthcare institutions in Lower Austria. Additionally, the survey link was distributed by the Austrian Association for Palliative Care (Österreichische Palliativgesellschaft, OPG) whose members are located throughout Austria and are predominantly PC physicians.

An initial email and a reminder email were sent to all relevant facilities. The survey period spanned from 15 November 2022 to 27 February 2023, coinciding with the first year of the legalization of AS in Austria.

### Data collection

Data were collected through a voluntary online questionnaire distributed to physicians practicing in hospitals across Lower Austria under the LGA and members of the OPG. The questionnaire was completed anonymously with data collected and stored securely on a dedicated, team-accessible website, following strict privacy guidelines.

### Study population

This study engaged two main groups: PC physicians and non-PC physicians, with the latter divided into specialty-based subgroups. The PC physicians were defined as those actively working in the field of PC, which includes settings such as PC wards, PC consultant teams, mobile PC teams, home care teams and hospices. In contrast, non-PC physicians were categorized as those not working in any of these specific PC settings. Participants were required to be doctors affiliated with LGA hospitals in Lower Austria or members of the OPG, aged 18 years or older and proficient in German.

Regarding training in PC, physicians employed by the LGA receive varying levels of training depending on their role and stage of career. Physicians in training within the LGA are required to attend a 4‑h compulsory lecture on the basics of PC. Beyond this foundation lecture, further PC training is typically obtained by physicians who pursue additional qualifications, such as a diploma or specialization in PC. These advanced training programs cover in-depth the knowledge and skills necessary for PC practice.

### Data analysis

Data collection was conducted using Findmind® (St. Gallen, SG, Switzerland), with the final update on 27 February 2023. Descriptive statistics were used to compare knowledge levels between PC and non-PC physicians.

Descriptive statistics were used to summarize the demographic characteristics and responses to survey questions. Inferential statistics were applied to compare knowledge, perceptions and practices between PC physicians and non-PC physicians and χ^2^-tests were conducted for categorical variables to identify significant differences in proportions, while independent samples t‑tests were used for continuous variables where applicable. A *p*-value of less than 0.05 was considered statistically significant.

The data were processed in Excel for tables and graphics, while IBM SPSS Statistics (Version 27.0, SPSS Inc., Armonk, NY, USA) was used for analyzing knowledge-based questions.

## Results

### Response rate

The questionnaire link was sent to 4000 medical doctors through the LGA and to 446 members of the OPG. The overall response rate was 6.9% (*n* = 308). The completion rate was 5.0% (*n* = 223) of the physicians contacted, with 39.9% (*n* = 89) being PC physicians and 60.1% (*n* = 134) being non-PC physicians.

### Demographics

**Table 1:** Demographics of participants

Number of participants = 223		PC physicians	Non-PC physicians
Age	18–25 years	0 (0.0%)	1 (0.7%)
	26–30 years	3 (3.4%)	14 (10.4%)
	31–40 years	11 (12.4%)	36 (26.9%)
	41–50 years	42 (47.2%)	37 (27.6%)
	51–60 years	18 (20.2%)	28 (20.9%)
	Over 61 years	15 (16.9%)	18 (13.4%)
Gender	Male	24 (27.0%)	59 (44.0%)
	Female	65 (73.0%)	75 (56.0%)
Work experience	Less than 5 years	11 (12.4%)	27 (20.1%)
	6–10 years	18 (20.2%)	26 (19.4%)
	11–15 years	20 (22.5%)	17 (12.7%)
	16–20 years	10 (11.2%)	18 (13.4%)
	More than 21 years	30 (33.7%)	46 (34.3%)
Palliative medicine training courses (multiple answers possible)	Palliative care diploma from the Austrian Medical Association	66 (74.2%)	24 (17.9%)
	Interprofessional basic course	47 (52.8%)	10 (7.5%)
	Advanced course	33 (37.1%)	6 (4.5%)
	Master’s course in palliative care	14 (15.7%)	2 (1.5%)
	Specialization in palliative care	43 (48.3%)	2 (1.5%)
	None	0 (0.0%)	99 (73.9%)
	Other	3 (3.3%)	5 (3.7%)
Medical subfields	General medicine	59 (66.3%)	39 (29.1%)
	Internal medicine	27 (30.3%)	38 (28.4%)
	Anesthesia and intensive care medicine	12 (13.5%)	27 (20.1%)
	Surgery	1 (1.1%)	15 (11.2%)
	Gynecology	0 (0.0%)	7 (5.2%)
	Neurology	1 (1.1%)	8 (6.0%)
	Orthopedics and traumatology	0 (0.0%)	5 (3.7%)
	Psychiatry	2 (2.2%)	9 (6.7%)
	Radiation oncology	0 (0.0%)	4 (3.0%)
	Pediatrics	1 (1.1%)	2 (1.5%)
	Pneumology	0 (0.0%)	3 (2.2%)
	Internal intensive care medicine	0 (0.0%)	5 (3.7%)
	Physical medicine	0 (0.0%)	2 (1.5%)

The demographics of the study participants are summarized in Table 1 and highlight distinct profiles between PC and non-PC physicians. Both groups shared a median age range of 41–50 years and a work experience range of 11–15 years. Gender distribution differed, with 73.0% of PC physicians being female compared to 56.0% among non-PC physicians. Training in PC was notably more common among PC physicians, with 74.2% holding a PC diploma, while 73.9% of non-PC physicians reported no palliative training. Subspecialty distributions also varied, with PC physicians predominantly in general medicine (66.3%), while non-PC physicians were more evenly distributed across multiple specialties.

### Questionnaire results (Table 2)

#### Knowledge about palliative sedation therapy

**Table 2:** Questionnaire results—Knowledge-based questions

Topic	Choice of answers	PC physicians	Non-PC physicians	*p*-value
**Knowledge-based questions**				
*Palliative sedation therapy*				
What is palliative sedation? (multiple answers possible)	–	*n* = 89	*n* = 134	–
	Active euthanasia of the patient in case of unbearable suffering	1 (1.1%)	1 (0.7%)	ns
	Relief from unbearable suffering	66 (74.2%)	102 (76.1%)	ns
	Assistance in suicide	0 (0.0%)	0 (0.0%)	ns
	Administration of medication to control symptoms	74 (83.1%)	122 (91.0%)	ns
	An alternative to euthanasia	10 (11.2%)	13 (9.7%)	ns
	Last resort for symptom control	73 (82.0%)	56 (41.8%)	< 0.001
Which drugs are used for palliative sedation according to guidelines? (multiple answers possible)	–	*n* = 89	*n* = 134	–
	Morphine	30 (33.7%)	94 (70.1%)	< 0.001
	Midazolam	87 (97.8%)	104 (77.6%)	< 0.001
	Levomepromazine	21 (23.6%)	15 (11.2%)	0.016
	Hydromorphone	12 (13.5%)	63 (47.0%)	< 0.001
	Propofol	50 (56.2%)	51 (38.1%)	0.009
	Diphenhydramine	2 (2.2%)	6 (4.5%)	ns
What is an indication for palliative sedation? (multiple answers possible)	–	*n* = 89	*n* = 134	–
	Agitated delirium in the terminal phase	59 (66.3%)	95 (70.9%)	ns
	Refractory dyspnea	79 (88.8%)	116 (86.6%)	ns
	Pain refractory to therapy	77 (86.5%)	118 (88.1%)	ns
	Massive bleeding	52 (58.4%)	40 (29.9%)	< 0.001
	Asphyxia	38 (42.7%)	43 (32.1%)	ns
	Desire for death	4 (4.5%)	4 (3.0%)	ns
	Existential suffering	61 (68.5%)	53 (39.6%)	< 0.001
	Refractory emesis	61 (68.5%)	41 (30.6%)	< 0.001
	Status epilepticus in the palliative setting	68 (76.4%)	88 (65.7%)	ns
Who is allowed to perform palliative sedation therapy?	–	*n* = 89	*n* = 134	–
	Physicians	89 (100%)	132 (98.5%)	ns
	Nurses	9 (10.1%)	18 (13.4%)	ns
	Paramedics	0 (0.0%)	1 (0.8%)	ns
	Relatives	3 (3.4%)	3 (2.2%)	ns
	Pharmacists	0 (0.0%)	0 (0.0%)	ns
*Assisted suicide*				
What are the requirements for approval of assisted suicide? (multiple answers possible)	–	*n* = 89	*n* = 134	–
	Incurable, fatal disease	80 (89.9%)	121 (90.3%)	ns
	Decision-making ability, adult	88 (98.9%)	120 (89.6%)	0.006
	Permanent, free and self-determined decision	78 (87.6%)	121 (90.3%)	ns
	Serious, long-term illness with persistent symptoms	57 (64.0%)	84 (62.7%)	ns
	Temporary serious illness	0 (0.0%)	1 (0.7%)	ns
What conditions must be met according to the law to carry out assisted suicide? (multiple answers possible)	–	*n* = 89	*n* = 134	–
	Visiting two different doctors (1× palliative doctor)	87 (97.8%)	124 (92.5%)	ns
	Consult a notary	71 (79.8%)	74 (55.2%)	< 0.001
	Consult a lawyer	17 (19.1%)	24 (17.9%)	ns
	Time lapse of 12 weeks until carrying out	81 (91.0%)	91 (67.9%)	< 0.001
	Psychological assessment (if required)	79 (88.8%)	98 (73.1%)	0.006
	Collection of the drug from the public pharmacy	80 (89.9%)	88 (65.7%)	< 0.001
	Collection of the drug from the doctor’s pharmacy	5 (5.6%)	9 (6.7%)	ns
How long is the legal waiting period before assisted suicide can be carried out?	–	*n* = 89	*n* = 134	–
	12 weeks	73 (82.0%)	98 (73.1%)	ns
	12 months	6 (6.7%)	1 (0.7%)	0.017
	2 weeks (in terminal phase)	63 (70.8%)	55 (41.0%)	< 0.001
	2 months (in terminal phase)	2 (2.2%)	3 (2.2%)	ns
	6 weeks (in terminal phase)	8 (9.0%)	19 (14.2%)	ns
	6 months	3 (3.4%)	2 (1.5%)	ns
Who is performing the assisted suicide?	–	*n* = 89	*n* = 134	–
	Physicians	6 (6.7%)	65 (48.5%)	< 0.001
	Pharmacists	0 (0.0%)	2 (1.5%)	ns
	Relatives	3 (3.4%)	9 (6.7%)	ns
	Nurses	0 (0.0%)	6 (4.5%)	ns
	Patients	80 (89.9%)	72 (53.7%)	< 0.001
What topics are covered during consultation about assisted suicide? (multiple answers possible)	–	*n* = 89	*n* = 134	–
	Possible treatment or action alternatives	83 (93.3%)	128 (95.5%)	ns
	The dosage of the drug and the necessary concomitant medication	80 (89.9%)	100 (74.6%)	0.005
	Method of taking the drug	84 (94.4%)	120 (89.6%)	ns
	Effects and possible complication of taking the drug	84 (94.4%)	116 (86.6%)	ns
	Death decree act that life-saving treatment can be refused as well as information about healthcare proxy	76 (85.4%)	107 (79.9%)	ns
	Reference to concrete offers for a psychotherapeutic discussion as well as for suicide prevention advice	64 (71.9%)	102 (76.1%)	ns
	A reference to any other counselling services that may be useful in the specific case	73 (82.0%)	93 (69.4%)	0.040
	Compulsory psychological/psychiatric interview	11 (12.4%)	63 (47.0%)	< 0.001
Permitted actions by healthcare professionals while preparing lethal medication? (multiple answers possible)	–	*n* = 89	*n* = 134	–
	Preparation of the infusion	72 (80.9%)	90 (67.2%)	0.030
	Dissolve the powder for oral use	69 (77.5%)	92 (68.7%)	ns
	Insert needle for an intravenous application	76 (85.4%)	94 (70.1%)	0.010
	Turning on the infusion	2 (2.2%)	30 (22.4%)	< 0.001
	Document the last thoughts of the person desiring death	67 (75.3%)	84 (62.7%)	ns

*n* number of participants, *ns* not significant, *PC* palliative care

##### What is palliative sedation? (multiple answers possible)

Most PC (83.1%) and non-PC (91.0%) physicians agreed that PST involves the administration of medication to control symptoms, with no significant differences. Both groups also agreed on relief from unbearable suffering (PC: 74.2%, non-PC: 76.1%); however, significantly more PC physicians (82%) defined PST as the last resort for symptom control compared to non-PC physicians (41.8%, *p* < 0.001). Few physicians viewed PST as active euthanasia, and none defined it as assistance in suicide.

##### Which drugs are used for palliative sedation according to guidelines? (multiple answers possible)

In PST, significantly more PC physicians use midazolam (97.8% vs. 77.6%, *p* < 0.001), propofol (56.2% vs. 38.1%, *p* = 0.009) and levomepromazine (23.6% vs. 11.2%, *p* = 0.016), while non-PC physicians prefer morphine (70.1% vs. 33.7%, *p* < 0.001) and hydromorphone (47% vs. 13.5%, *p* < 0.001). There was no significant difference in the use of diphenhydramine.

##### What is an indication for palliative sedation? (multiple answers possible)

Both PC and non-PC physicians commonly cited refractory dyspnea and pain refractory to therapy as indications for PST, with no significant differences; however, PC physicians more often cited massive bleeding (58.4% vs. 29.9%, *p* < 0.001), existential suffering (68.5% vs. 39.6%, *p* < 0.001) and refractory emesis (68.5% vs. 30.6%, *p* < 0.001). Status epilepticus showed no significant difference, while asphyxia and desire for death were less frequently mentioned.

##### Who is allowed to perform PST?

All PC physicians and 98.5% of non-PC physicians agreed that physicians can administer PST. A minority believed nurses are allowed (10.1% PC, 13.4% non-PC), while few mentioned relatives (3.4% PC, 2.2% non-PC) or paramedics (0.0% PC, 0.8% non-PC). No group considered pharmacists as administrators. There were no significant differences between the groups.

#### Knowledge about assisted suicide

(Table 2)

##### What are the requirements for approval of AS? (multiple answers possible)

The PC physicians most frequently cited decision-making ability, adult (98.9%), incurable, fatal disease (89.9%), and permanent, free and self-determined decision (87.6%) as indications for AS. Non-PC physicians similarly cited incurable, fatal disease and permanent, free and self-determined decision (both 90.3%), and decision-making ability, adult (89.6%). The only significant difference was PC physicians more often acknowledging decision-making ability, adult (*p* = 0.006).

##### What conditions must be met according to the law to carry out AS? (multiple answers possible)

A consultation with two doctors, including a PC physician, was recognized by 97.8% of PC-trained and 92.5% of non-PC physicians. Legal requirements were better understood by PC-trained physicians, including consulting a notary (79.8% vs. 55.2%, *p* < 0.001), the 12-week waiting period (91% vs. 67.9%, *p* < 0.001) and psychological assessment if needed (88.8% vs. 73.1%, *p* = 0.006). Additionally, 89.9% of PC-trained physicians knew the drug must be collected from a public pharmacy, compared to 65.7% of non-PC physicians (*p* < 0.001).

##### How long is the legal waiting period before AS can be carried out?

Regarding waiting periods for AS, 82% of PC and 73.1% of non-PC physicians agreed on a 12-week period, with no significant difference; however, 70.8% of PC physicians supported a 2-week period in terminal phases, compared to 41.0% of non-PC physicians (*p* < 0.001). A 12-month waiting period was favored by 6.7% of PC physicians, significantly more than 0.7% of non-PC physicians (*p* = 0.017). No differences were found for other waiting periods.

##### Who is performing AS?

Among PC physicians, 89.9% believe patients perform AS, compared to 53.7% of non-PC physicians (*p* < 0.001). Conversely, 48.5% of non-PC physicians think physicians perform AS, compared to 6.7% of PC physicians (*p* < 0.001). Smaller percentages in both groups mentioned relatives, nurses, or pharmacists, with no significant differences for these roles.

##### What topics are covered during consultation about AS? (multiple answers possible)

During AS consultations PC physicians frequently discussed medication effects and complications (94.4%), administration methods (94.4%), alternative treatments (93.3%), dosage and concomitant medication (89.9%, *p* = 0.005) and living wills (85.4%). Non-PC physicians similarly addressed alternative treatments (95.5%) and administration methods (89.6%) but emphasized compulsory psychological interviews more (48.0% vs. 12.4%, *p* < 0.001). The PC physicians more often referred patients to other counselling services (82.0% vs. 69.4%; *p* = 0.040).

##### Permitted actions by health care professionals while preparing lethal medication? (multiple answers possible)

The PC physicians were more likely to consider needle insertion (85.4% vs. 70.1%, *p* = 0.010) and infusion preparation (80.9% vs. 67.2%, *p* = 0.030) permissible, while non-PC physicians more often accepted turning on the infusion (22.4% vs. 2.2%, *p* < 0.001). No significant differences were found for dissolving powder for oral use or documenting last thoughts.

#### Perception and attitude questions

(Table 3)

**Table 3:** Questionnaire results—Perception and attitude questions

Topic	Choice of answers	PC physicians	Non-PC physicians	*p*-value
*Perception and attitude questions*				
How do you see the discussion by PC professionals in relation to AS? (multiple answers possible)	–	*n* = 89	*n* = 134	–
	Can prevent AS	42 (47.2%)	32 (23.9%)	< 0.001
	Can show alternatives to AS	84 (94.4%)	123 (91.8%)	ns
	Has no influence	3 (3.4%)	9 (6.7%)	ns
Who should provide information to patients about PST? (multiple answers possible)	–	*n* = 89	*n* = 134	–
	PC physicians	88 (98.9%)	125 (93.3%)	ns
	Anesthesiologists	57 (64.0%)	89 (66.4%)	ns
	General practitioners	51 (57.3%)	93 (69.4%)	ns
	Nurses	28 (31.5%)	45 (33.6%)	ns
	Specialists of all disciplines	35 (39.3%)	79 (59.0%)	0.006
	Other medical specialties	4 (4.5%)	6 (4.5%)	ns
What procedures do you consider as alternatives to AS (multiple answers possible)	–	*n* = 89	*n* = 134	–
	PST	57 (64.0%)	96 (71.6%)	ns
	Voluntary abstinence from liquid and food intake	41 (46.0%)	60 (44.8%)	ns
	Advance directives	15 (16.9%)	20 (14.9%)	ns
	Power of attorney	2 (2.2%)	7 (5.2%)	ns
	Suicide	55 (61.8%)	85 (63.4%)	ns
Who should inform patients about the option of AS? (multiple answers possible)	–	*n* = 89	*n* = 134	–
	Self-help groups	26 (29.2%)	56 (41.8%)	ns
	PC physicians	44 (49.4%)	113 (84.3%)	< 0.001
	Anesthesiologists	19 (21.3%)	56 (41.8%)	ns
	Specialists	30 (33.7%)	73 (54.5%)	< 0.001
	General practitioners	42 (47.2%)	92 (68.7%)	< 0.001
	Nurses	10 (11.2%)	25 (18.7%)	ns
	Lawyers	28 (31.5%)	26 (19.4%)	ns
	Family of the patient	4 (4.5%)	9 (6.7%)	ns
	Nobody of the mentioned groups	25 (28.1%)	7 (5.2%)	< 0.001
Do you feel qualified enough to inform patients about AS?	–	*n* = 89	*n* = 134	–
	Qualified and consolidated	22 (24.7%)	12 (9.0%)	< 0.001
	Qualified but additional support is needed	21 (23.6%)	27 (20.1%)	ns
	Not qualified	42 (47.2%)	94 (70.1%)	ns
Would you consider utilizing PST as a patient?	–	*n* = 89	*n* = 134	–
	Would use PST	66 (74.2%)	105 (78.4%)	ns
	Would not use PST	6 (6.7%)	13 (9.7%)	ns
	No answer	17 (19.1%)	16 (11.9%)	ns
Would you consider utilizing AS as a patient?	–	*n* = 89	*n* = 134	–
	Would use AS	16 (18%)	50 (37.3%)	ns
	Would not use AS	41 (46.1%)	49 (36.6%)	ns
	No answer	32 (36.0%)	34 (25.4%)	ns

*AS* assisted suicide, *n* number of participants, *ns* not significant, *PC* palliative care, *PST* palliative sedation therapy

##### How do you see the discussion by palliative care professionals in relation to AS? (multiple answers possible)

Regarding the consultation possibilities, 94.4% of PC physicians and 91.8% of non-PC physicians stated it “can show alternatives to AS.” Additionally, 47.2% of PC physicians and 23.9% of non-PC physicians believed it “can prevent AS.” Only 3.4% of PC physicians and 6.7% of non-PC physicians felt the consultation “has no influence.”

##### Who should provide information to patients about PST? (multiple answers possible)

Regarding who should inform patients about PST, 98.9% of PC physicians and 93.3% of non-PC physicians believe PC physicians should be responsible. Additionally, 64% of PC physicians and 66.4% of non-PC physicians think anesthesiologists should inform patients. For further results, see Table 3. No statistical significance could be observed between the two groups.

##### What procedures do you consider as alternatives to AS? (multiple answers possible)

Both PC and non-PC physicians most favored palliative sedation as an alternative to AS (64.0% and 71.6%, respectively), followed by voluntary abstinence from liquid and food intake (46.0% and 44.8%, respectively). Advance directives were also supported, though less frequently, while power of attorney was rarely favored. Suicide was considered irrelevant by most (PC: 61.8%, non-PC: 63.4%), with no significant differences between the groups.

##### Who should inform patients about the option of AS? (multiple answers possible)

Regarding who should inform patients about AS, 49.4% of PC physicians and 84.3% of non-PC physicians believe PC physicians should (*p* < 0.001). Additionally, 47.2% of PC physicians and 68.7% of non-PC physicians think general practitioners should (*p* < 0.001). Specialists were suggested by 33.7% of PC physicians and 54.5% of non-PC physicians (*p* < 0.001). For all other results, see Table 3.

##### Do you feel qualified enough to inform patients about AS?

Nearly half of PC physicians (47.2%) felt not qualified for AS, while 24.7% felt qualified and consolidated and 23.6% needed additional support. Among non-PC physicians, 70.1% felt not qualified, 20.1% needed support and only 9.0% felt qualified and consolidated. The PC physicians were significantly more likely to feel qualified (*p* < 0.001).

##### Would you consider utilizing PST as a patient?

Regarding the use of PST for themselves, 74.2% of PC physicians and 78.4% of non-PC physicians indicated they would use it. No statistical significance could be observed between the two groups.

##### Would you consider utilizing AS as patient?

Regarding who would consider AS, 46.1% of PC physicians answered “would not,” 36% gave “no answer,” and 18% answered “would.” Among non-PC physicians, 37.3% answered “would not,” 36.6% answered “would,” and 25.4% gave “no answer.”

#### Experience and practice questions

**Table 4:** Questionnaire results—Experience and practice questions

Topic	Choice of answers	PC physicians	Non-PC physicians	*p*-value
*Experience and practice questions*				
How often have you performed PST?	–	*n* = 89	*n* = 134	–
	Never	13 (14.6%)	62 (46.3%)	< 0.001
	1–10 times	32 (36.0%)	31 (23.1%)	0.047
	11–20 times	14 (15.7%)	7 (5.2%)	0.010
	More than 20 times	29 (32.6%)	33 (24.6%)	ns
Which drugs do you use most often for PST? (multiple answers possible)	–	*n* = 89	*n* = 134	–
	Morphine	26 (29.2%)	81 (50.5%)	< 0.001
	Midazolam	85 (95.5%)	73 (54.4%)	< 0.001
	Levomepromazine	4 (4.5%)	3 (2.2%)	ns
	Hydromorphone	5 (5.6%)	23 (17.2%)	0.022
	Propofol	19 (21.3%)	24 (17.9%)	ns
	Diphenhydramine	0 (0.0%)	1 (0.8%)	ns
Do you have access to supervision?	–	*n* = 89	*n* = 134	–
	Yes	77 (86.5%)	69 (51.5%)	< 0.001
	No	8 (9.0%)	42 (31.3%)	< 0.001
	No information	3 (3.4%)	23 (17.2%)	< 0.001

*n* number of participants, *ns* not significant, *PC* palliative care, *PST* palliative sedation therapy

The PST is used significantly more by PC physicians. Midazolam is the most common drug, though opioids and diphenhydramine are sometimes incorrectly used, more frequently by non-PC physicians. Supervision is more accessible to PC physicians (86.5%) compared to non-PC physicians (51.5%). Further details are described in Table 4.

## Discussion

The results of our survey provide valuable insights into the knowledge, perceptions, attitudes and experiences of medical doctors towards PST and AS in Austria. Our findings show significant differences between PC physicians and non-PC physicians in their understanding and practices related to these areas.

In terms of PST, both PC and non-PC physicians primarily defined PST as administering medication to control symptoms and relieving unbearable suffering; however, a notable difference was observed in defining PST as the “last resort for symptom control,” with significantly more PC physicians holding this view, indicating their deeper familiarity with its use in clinical practice. This finding suggests that PC physicians, through their specialized training and practice, are more attuned to the complexities and ethical considerations of PST. The high use of guideline-recommended drugs, such as midazolam by PC physicians aligns with established guidelines [11] while the substantial use of non-recommended drugs, such as morphine by non-PC physicians suggests a gap in adherence to PST guidelines. These discrepancies suggest improving education and adherence to PST guidelines to ensure optimal patient care and avoid potential complications arising from the use of inappropriate medications.

The use of morphine as a sedative in PC, as described in our manuscript, warrants further discussion. Morphine is an opioid primarily indicated for the palliation of pain and dyspnea but it is not a sedative and does not reliably induce sustained sedation. While it may be used adjunctively in the context of refractory symptoms, morphine alone is not appropriate for achieving palliative sedation. As highlighted by Kompanje et al. [12] PST should be managed with benzodiazepines, such as midazolam, which are specifically indicated for symptoms, such as terminal agitation and delirium. The widespread use of morphine for sedation, as noted in our study and others, reflects a gap in adherence to evidence-based guidelines, potentially leading to suboptimal management of refractory symptoms.

Little is known about the attitude towards AS in Austria. In 2016, a nationwide survey in Austria examined attitudes towards AS and euthanasia among 493 care-dependent adults aged 50+ years in private households. Approximately 25% supported the availability of AS and euthanasia or would hypothetically consider using them. The use of AS was more accepted by those living in urban areas, distrustful of physicians, with active suicide ideation or a strong fear of dying. Acceptance of euthanasia was associated with living alone, low religiosity and fear of dying. There is a need for expanded community-based psychosocial care to address psychological distress and end of life fears among this population [13].

Regarding the approval of AS, both PC and non-PC physicians largely agreed on the key conditions, including incurable, fatal diseases and a permanent, free and self-determined decision; however, PC physicians emphasized decision-making ability and adulthood more, reflecting their stricter criteria for AS. This difference might be attributed to PC physicians’ more frequent engagement with end of life care and their nuanced understanding of patient autonomy and decision-making capacity. The survey also revealed that PC physicians were more informed about procedural requirements for AS, such as consulting a notary, the 12-week waiting period, psychological assessments and collecting the drug from a public pharmacy. This indicates a need for improved education on legal requirements among non-PC physicians, ensuring they are well-informed about the procedural safeguards and ethical implications associated with AS.

Regarding experiences and attitudes towards AS, a recent study surveyed 280 PC and hospice nurses to explore their experiences and attitudes towards AS after the first year of implementation. Over 61% had cared for a patient who expressed a wish for AS. More nurses supported AS (50%) than opposed it (32%). Older age, religiosity and prior experience with patients requesting AS were associated with greater reluctance towards AS [14].

The overwhelming consensus among both PC and non-PC physicians is that PC consultations are effective in presenting alternatives to AS; however, PC physicians are more likely to believe that these consultations can actively prevent AS, suggesting that their specialized training provides them with more confidence in the impact of consultations. Based on our findings, we consider consultations in providing patients with comprehensive end of life care options, emphasizing the role of thorough and compassionate communication in end of life decision-making as important. The small percentage of physicians who believe consultations have no influence underlines the general belief in the value of these discussions, reinforcing the need for structured and well-supported consultation processes.

Regarding who should inform patients about PST, both groups overwhelmingly agreed that PC physicians should take on this role. There was also significant support for anesthesiologists and general practitioners. The varied responses for other specialists and nurses could suggest a need for clearer guidelines on the roles of different healthcare providers in this communication process. Establishing clear protocols and training programs for various healthcare providers can enhance the consistency and quality of patient education about PST.

Both PC and non-PC physicians favored PST and voluntary abstinence from food and liquid as alternatives to AS, reflecting a preference for approaches that alleviate suffering without active intervention in death. Advance directives are also valued, though less prominently. The minimal support for the power of attorney suggests it is not seen as a direct alternative to AS. The consensus that suicide plays no role underlines a shared ethical stance against it. The results of our survey value the importance of promoting PC options and clear communication about end of life choices [15]. Ensuring that patients and their families are well-informed about these alternatives can aid in making informed and ethically sound decisions.

Our results indicate that both PC and non-PC physicians require more information and education about AS. Non-PC physicians need enhanced education about PST in general. Despite PC physicians having more experience, gaps in adherence to guidelines, such as the incorrect use of medications for PST, suggest that even they need continued education and reinforcement of best practices. The disparity in access to supervision, with PC physicians having significantly better access compared to non-PC physicians, calls for better support structures. Improved access to supervision and targeted educational programs for non-PC physicians could bridge these gaps, ensuring all healthcare providers are well-equipped to deliver high-quality PC and appropriately manage end of life options.

### Limitations and strengths

A key limitation of our study is the low response rate, which may reflect limited interest in the topics and potentially bias the findings, particularly among non-PC physicians. This underlines the challenge of engaging busy medical professionals in survey-based research. Despite this, the study provides valuable insights into knowledge gaps and attitudes toward PC and AS, highlighting the need for targeted educational interventions and improved training and support structures.

As the survey was anonymous and did not include a question about the respondents’ geographic location, it was not possible to determine the regional distribution of the responses. This limits our ability to draw conclusions about whether the data predominantly represents physicians from Lower Austria or from other regions of Austria.

A further limitation of our study is the lack of a detailed analysis examining the influence of years of PC-specific experience on knowledge levels. While our survey included information on general work experience, it did not specifically capture the duration of PC practice and we did not statistically analyze the minimum years of experience required to demonstrate significant knowledge differences.

One noteworthy aspect of our study is the potential for response bias. Given the low response rate, it is likely that physicians who chose to participate were those with a particular interest or involvement in the topics of AS and PST. Despite this presumed interest, our findings revealed notable knowledge gaps among the respondents. This suggests that the overall knowledge within the broader population of physicians may be even lower. These findings highlight the urgent need for comprehensive education and training on AS and PST across all medical disciplines.

## Conclusion

Our survey reveals significant gaps in knowledge and adherence to guidelines for both PST and AS among PC and non-PC physicians. While PC physicians demonstrate higher training levels, errors in PST medication use persist and non-PC physicians require more comprehensive education on PST and PC. Limited access to supervision further highlights the need for enhanced support structures. Addressing these gaps through improved education and supervision is essential to ensure optimal patient care and increase physician confidence in managing sensitive end of life issues.

## Data Availability

The datasets used and/or analyzed during this study are available from the corresponding author on reasonable request. The data are not publicly available.

## Abbreviations

AS: Assisted suicide
COPD: Chronic obstructive pulmonary disease
LGA: State Health Agency (Landesgesundheitsagentur)
OPG: Austrian Association for Palliative Care (Österreichische Palliativgesellschaft)
PC: Palliative care
PST: Palliative sedation therapy
StVfG: Assisted Dying Act (Sterbeverfügungsgesetz)
